# Needs Assessment Survey Identifying Research Processes Which may be Improved by Automation or Artificial Intelligence: ICU Community Modeling and Artificial Intelligence to Improve Efficiency (ICU-Comma)

**DOI:** 10.1177/08850666211064844

**Published:** 2021-12-13

**Authors:** Vincent I. Lau, Alexandra Binnie, John Basmaji, Nadia Baig, Dawn Opgenorth, Saoirse Cameron, Katie O’Hearn, Ellen McDonald, Janek Senaratne, Wendy Sligl, Danny J. Zuege, Oleksa Rewa, Sean M. Bagshaw, Jennifer Tsang

**Affiliations:** 1 12357 University of Alberta and Alberta Health Services, Edmonton, Alberta, Canada; 2 60407 William Osler Health System, Brampton, ON, Canada; 3 6221 Western University, London, ON, Canada; 4 10033 London Health Sciences Center, London, ON, Canada; 5 274065 Children’s Hospital of Eastern Ontario Research Institute, Ottawa, ON, Canada; 6 3710 McMaster University, Hamilton, ON, Canada; 7 3158 University of Alberta, Edmonton, Alberta, Canada; 8 70401 University of Calgary, Calgary, Alberta, Canada; 9 Alberta Health Services, Alberta, Canada; 10 37195 Niagara Health, St. Catharines, Ontario, Canada

**Keywords:** automation, artificial intelligence, critical care, research, efficiency, needs assessment, survey

## Abstract

**Background:**

Critical care research in Canada is conducted primarily in academically-affiliated intensive care units with established research infrastructure, including research coordinators (RCs). Recently, efforts have been made to engage community hospital ICUs in research albeit with barriers. Automation or artificial intelligence (AI) could aid the performance of routine research tasks. It is unclear which research study processes might be improved through AI automation.

**Methods:**

We conducted a cross-sectional survey of Canadian ICU research personnel. The survey contained items characterizing opinions regarding research processes that may be amenable to AI automation. We distributed the questionnaire via email distribution lists of 3 Canadian research societies. Open-ended questions were analyzed using a thematic content analysis approach.

**Results:**

A total of 49 survey responses were received (response rate: 8%). Tasks that respondents felt were time-consuming/tedious/tiresome included: screening for potentially eligible patients (74%), inputting data into case report forms (65%), and preparing internal tracking logs (53%). Tasks that respondents felt could be performed by AI automation included: screening for eligible patients (59%), inputting data into case report forms (55%), preparing internal tracking logs (51%), and randomizing patients into studies (45%). Open-ended questions identified enthusiasm for AI automation to improve information accuracy and efficiency while freeing up RCs to perform tasks that require human interaction. This enthusiasm was tempered by the need for proper AI education and oversight.

**Conclusions:**

There were balanced supportive (increased efficiency and re-allocation of tasks) and challenges (informational accuracy and oversight) with regards to AI automation in ICU research.

## Background

Critical care research in Canada is conducted primarily in academic hospital intensive care units (ICUs) affiliated with universities. Most community hospitals have barriers to participation in research due to a lack of hospital culture supporting research; interested/dedicated clinicians; integrated electronic medical records (EMR)/health systems; funding for research; preexisting research infrastructure (eg, research personnel).^1–3^ Moreover, the majority of research centers have full-time research staff, including research coordinators (RCs), who are vital for research program functioning.^1^ Although efforts have been made to increase clinical research engagement amongst community hospital ICUs, many community hospitals lack dedicated research infrastructure as well as access to RCs. Moreover, the cost of RC support has been identified as a major barrier to community hospital research engagement.^1,4^

Increasing community ICUs participation (which provides ∼65% of ICU care in Canada)^1^ in multi-center trials would improve recruitment nationally, shorten the duration of clinical trials and increase the quality of evidence as well. It would also improve the generalizability of study results and accelerate knowledge translation^1^. Strategies to facilitate community ICU involvement in research are required. Incorporating artificial intelligence (AI) automation to perform basic research tasks could reduce research costs, making research more feasible in both community and academic ICUs. Moreover, using AI programs may also increase research efficiencies for research in general^5,6^ even outside of the ICU setting (eg, all areas of medicine). Contiguous, universal research programs are needed to follow complex research patients from admission to hospital, throughout their stay in any department, and until their discharge, and even beyond (eg, follow-up).

Automation or AI program could also allow RCs at academic hospitals to remotely support smaller community ICUs with insufficient patient volumes to justify in-house research coordination. This increase in RC productivity could decrease the costs of clinical research participation, thereby lowering a key barrier to entry for community ICUs.

Many RC tasks have inherent inefficiencies that might be improved by identifying processes that could be automated and/or assisted by AI, thereby enabling RCs to focus on tasks that require human interaction. Other areas that have been aided by AI including manufacturing,^7^ aviation,^8^ business economics,^9^ and even healthcare.^10^

The purpose of this study was to perform a needs assessment survey of Canadian critical care researchers and research support staff to identify research study processes that are perceived to be time-consuming, tedious, and/or tiresome to perform as well as which research study processes (for both academic and community research), could be most amenable to AI automation, both within ICU and outside of it.

## Methods

### Research Question

For Canadian critical care researchers and research support staff from 3 critical care research societies [population], what are the most time-consuming, tedious, or tiresome tasks which could be enhanced by AI automation [intervention] compared to traditional RC/research assistant (RA) processes/tasks/workflow [comparator], that could: (1) increase RC/RA efficiency; and (2) lead to a more efficient implementation of research programs in ICU settings without preexisting research infrastructure [outcomes]?

### Working Definitions

We used Canadian Institute of Health Information (CIHI)^11^ and Statistics Canada^12^ definitions for center population size (small: 1000-29 999 residents, medium: 30 000-199 999 residents, large: >200 000 residents). We used CIHI definitions for academic (confirmed teaching status by the Canadian government) versus community hospitals^13^; community hospital was defined as a hospital that provides a range of services to a local community, is led by community-based health professionals, and provides inpatient beds.^14^

### Questionnaire Development

We conducted a cross-sectional survey of RCs, RAs, investigators (physicians), data management personnel, ethics board members, administrative personnel, clinical information systems personnel, patient/family research partners, project managers, and research graduate students (masters and PhD). We characterized attitudes and opinions regarding time-consuming/tedious/tiresome research processes/tasks and which tasks would be amenable to AI automation.

Survey development methodology followed the stages proposed by Burns et al^15^:

#### Item Generation and Reduction

All processes and tasks conducted by RCs, RAs, and researchers were identified in literature review.^16^ Selected co-authors (AB, NB, DO, WD, RMM, JT) provided feedback on the accuracy and relevance of the questions. Computer scientists also contributed to the development of the survey and program of research feasibility. Similar or duplicate questions were removed.

#### Pretesting and Clinical Sensibility Testing

The questionnaire was distributed to all study co-authors, which includes representatives of the target audiences. Pretesters reviewed the survey tool for comprehensibility, accuracy, comprehensiveness, and likelihood of yielding pertinent information regarding the attitudes and opinions of participants of the survey. Two stages of pretesting were conducted and the survey tool was modified according to the feedback provided.

#### Pilot Testing

Pilot testing of the survey tool was conducted by 8 RCs, RAs, and investigators from across Canada. Responses to open-ended comments were reviewed to identify whether questions were clear to participants.

### Questionnaire Administration

The questionnaire was created on the SurveyMonkey platform (SurveyMonkey Inc., San Mateo, California, USA: www.surveymonkey.com). The full questionnaire can be found in Supplemental Appendix 1. The questionnaire was distributed through the Canadian Community ICU Research Network (CCIRNet), Canadian Critical Care Trials Group (CCCTG) & Canadian Critical Care Research Coordinator Group (CCCRCG) via their respective mailing list servers. The questionnaire tool was open from October 13, 2020, to June 1, 2021. Three email reminders were sent via each list server.

### Data Analysis

Respondent characteristics were summarized using descriptive statistics (numbers and frequencies for nominal and ordinal variables; means and standard deviations or medians and interquartile ranges [IQR] for continuous variables). Questions related to ICU research processes/tasks and AI automation were summarized in aggregate across the entire sample. 95% confidence intervals [CI] were reported, where applicable, based on a significance level of 0.05. All survey responses were included, with adjustment of denominators for nonresponses.

Responses to open-ended questions were analyzed using a qualitative, thematic content analysis approach.^17,18^ Two investigators (VL and JB) independently reviewed all open-ended responses and developed a list of emerging themes and subthemes for each question. Themes and subthemes were discussed and identified until agreement was reached. A third investigator was available to resolve any disagreements. The identified themes and subthemes for related questions were then merged into a comprehensive list for each of the following topics: tasks researchers would feel comfortable performing with AI automation, positive and negative effects of AI automation, and general feelings/comments about AI automation.

All analysis was performed using Excel version 14.0.6 (Microsoft Corp, Redmond Washington, US), and SAS version 9.4 (Cary, North Carolina, US).

### Ethics

Delegated research ethics board (REB) review was obtained from the University of Alberta Research Ethics Office (Pro00103709) on Aug 27, 2020. All respondents provided implied consent by electronically participating in the survey.

## Results

### Study Baseline Characteristics

A total of 49 responses were received from the 610 members of the 3 societies, representing a calculated response rate of 8%. The baseline demographic characteristics of respondents are summarized in Table 1.

**Table 1.** Occupational Role, Level of Experience, Location of Work, Population Size, Primary Practice Setting of Research Respondents.

Role (n = 49)	Responses (%)
Clinical research coordinator	22 (45)
Physician research investigator	10 (20)
Clinical research assistant	7 (14)
Research project manager	4 (8)
Research data management assistant	2 (4)
Graduate student (MSc or PhD)	1 (2)
Non-physician research investigator	1 (2)
Research analyst	1 (2)
Research ethics service office members	1 (2)
Administrative research coordinator	0 (0)
Clinical information systems members	0 (0)
Patient and/or family research partner	0 (0)
Research administrative assistant	0 (0)
Research ethics board members	0 (0)
Level of experience (n = 49)	
0-5 years	14 (29)
6-10 years	18 (37)
11-15 years	7 (14)
> 20 years	6 (8)
Primary location of work (n = 48)	
Ontario (ON)	29 (60)
Quebec (QC)	9 (19)
Alberta (AB)	5 (10)
British Columbia (BC)	2 (4)
Manitoba (MB)	1 (2)
Nova Scotia (NS)	1 (2)
Saskatchewan (SK)	1 (2)
New Brunswick (NB)	0 (0)
Newfoundland and Labrador (NL)	0 (0)
Prince Edward Island (PEI)	0 (0)
Northwest Territories (NT)	0 (0)
Nunavut (NT)	0 (0)
Yukon (YT)	0 (0)
Population size (n = 49)	
Small population center (1000-29 999 residents)	2 (4)
Medium population center (30 000-199 999 residents)	6 (12)
Large population center (> 200 000 residents)	41 (84)
Primary practice setting for research (n = 49)	
Academic hospital (confirmed teaching status Canadian government)	41 (84)
Community hospital	6 (12)
Contract research organization	1 (2)
Unclear	1 (2)

The majority of respondents were research coordinators (22 of 49, 45%), with 6 to 10 years of experience (18 of 48, 37%). Most respondents worked in Ontario (29 of 48, 60%). Most respondents were from large centers (41 of 49, 84%) with academic affiliations (41 of 49, 84%).

### Identifying Time-Consuming, Tedious, and Tiresome Research Tasks

**Figure 1.**
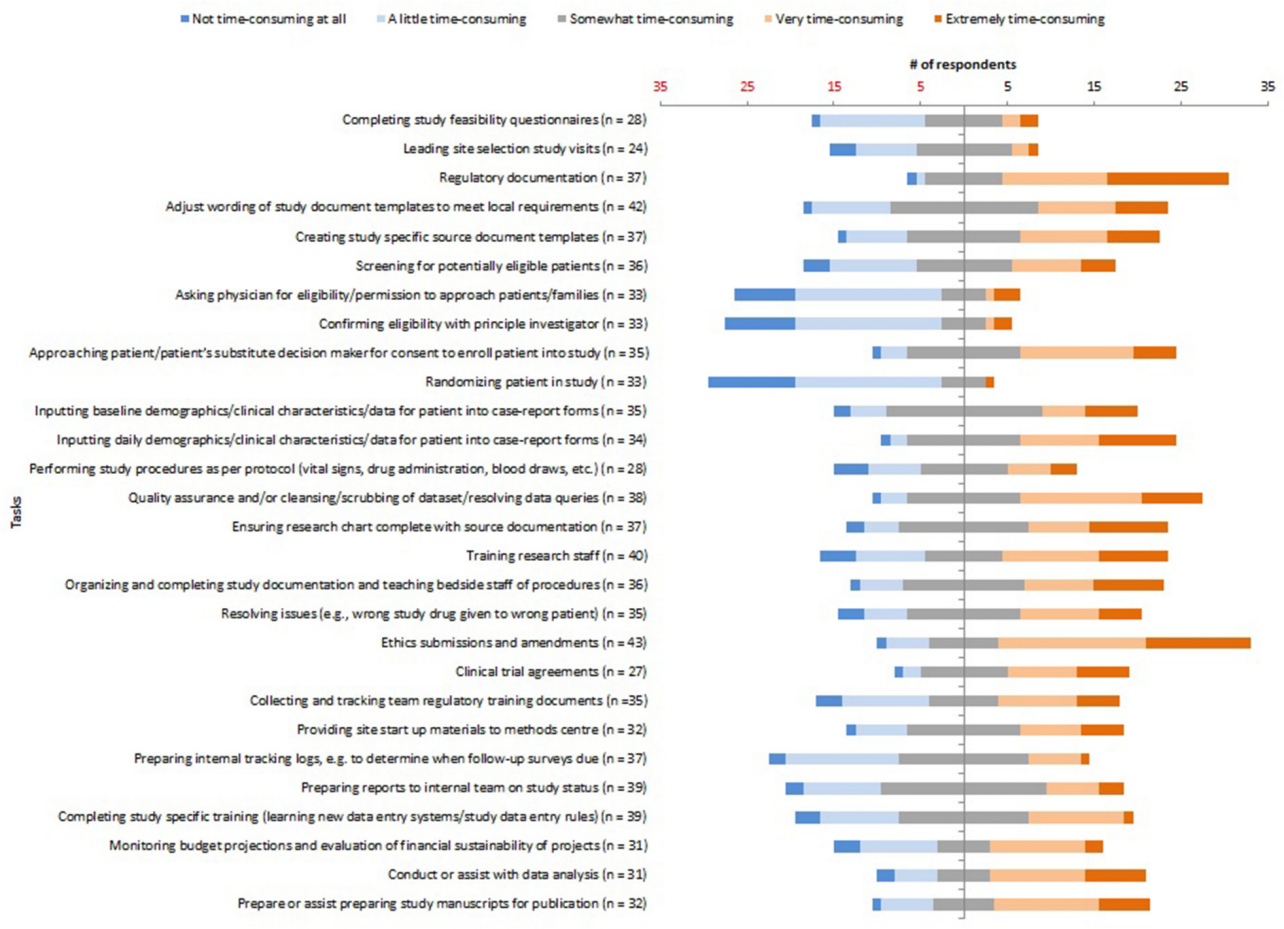
Tasks/processes considered time-consuming (Likert scale).

**Figure 2.**
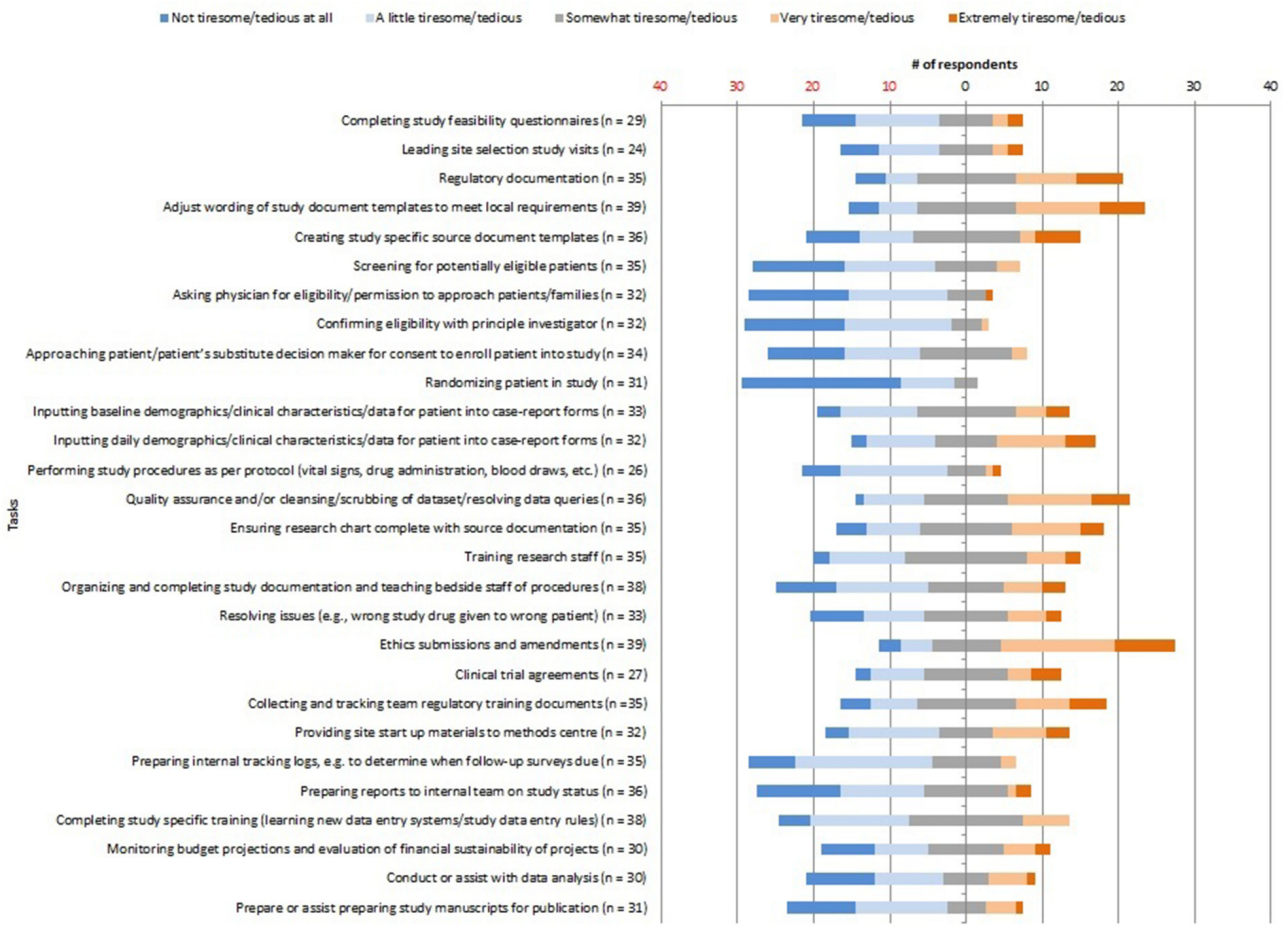
Tasks/processes considered tiresome/tedious (Likert scale).

Researcher roles for various tasks/processes are outlined in Supplemental Table 1. Researcher opinions about tasks considered time-consuming, tedious, or tiresome are shown in Figures 1 and 2.

The top 5 time-consuming tasks identified by respondents were creating study databases (100% extremely time-consuming), regulatory documentation (38% extremely and 32% very time-consuming), ethics submissions/amendments (28% extremely and 40% very time-consuming), inputting daily data (26% extremely and 26% very time-consuming), and ensuring research chart completion with source documentation (24% extremely and 19% very time-consuming).

The top 5 tedious/tiresome tasks were: ethics submissions/amendments (21% extremely and 38% very tedious/tiresome), regulatory documentation (17% extremely and 23% very tedious/tiresome), adjustment of wording of study documents to meet local requirements (15% extremely and 28% very tedious/tiresome), creating study-specific source documents (17% extremely, and 6% very tedious/tiresome), and quality assurance of data collection (14% extremely and 31% very tedious/tiresome).

### Tasks Amenable to AI Automation

Tasks identified by respondents as amenable to AI automation are presented in Table 2. The top 5 tasks were: screening for potentially eligible patients (74%), inputting baseline and daily data into case report forms (65%), preparing internal tracking logs (53%), collecting and tracking team regulatory training documents (49%), and preparing reports to internal team on study status (45%).

**Table 2.** Tasks Which Could be Aided by AI Automation (n = 49).

Task	Aided by AI *N* (%)	Comfort with AI *N* (%)
Screening for potentially eligible patients	36 (74)	29 (59)
Inputting baseline demographics, clinical characteristics, and data for patients into case-report forms (CRFs) Inputting daily demographics, clinical characteristics, and data for patients into CRFs	32 (65)	27 (55)
Preparing internal tracking logs, for example, to determine when follow-up surveys due	26 (53)	25 (51)
Collecting and tracking team regulatory training documents (eg, CVs, medical license, GCP training, TCPS2 training, privacy training, etc)	24 (49)	24 (49)
Preparing reports to internal team on study status	22 (45)	21 (43)
Randomizing patients in study	21 (43)	22 (45)
Quality assurance and/or cleansing/scrubbing of dataset/resolving data queries	21 (43)	20 (41)
Monitoring budget projections and ongoing evaluation of financial sustainability of research projects	21 (43)	13 (27)
Conduct or assist with data analysis	21 (43)	16 (33)
Creating study specific source document templates	20 (41)	19 (39)
Confirming eligibility with principal investigator	18 (37)	10 (20)
Ensuring research chart complete with source documentation	17 (35)	16 (33)
Adjust wording of study document templates (CDA, CTA, informed consents, study information letters, etc) to meet local requirements	17 (35)	19 (39)
Providing site start-up materials to methods center (eg, confirmation of training, CVs, medical license, contracts complete, delegation log, etc)	16 (33)	13 (27)
Organizing and completing study documentation for enrollment, randomization, and consent procedures to your list and informing/teaching nurse staff of study procedures and tests being done, and conducting follow-up bedside calls to ensure study procedures are being followed	15 (31)	14 (29)
Completing study feasibility questionnaires	14 (29)	15 (31)
Asking most responsible physician (MRP) for eligibility and permission to approach patients/families	12 (25)	10 (20)
Regulatory documentation (CDA, CTA, review or negotiate a budget, perform or provide input in the impact analysis of the study in coordination with supporting programs (pharmacy, laboratory, etc)	11 (22)	14 (29)
Completing study-specific training (eg, learning new data entry systems, learning each study data entry rules)	11 (22)	8 (16)
Training research staff	11 (22)	9 (18)
Ethics submissions and amendments	9 (18)	10 (20)
Clinical trial agreements	8 (16)	10 (20)
Performing study procedures as per protocol (vital signs, drug administration, venipuncture, blood draws, etc)	6 (12)	7 (14)
Resolving issues (eg, wrong study drug given to wrong patient)	5 (10)	3 (6)
Leading site selection study visits (inpatient or clinic area where study will be conducted, pharmacy, labs, etc)	5 (10)	6 (12)
Approaching patient or patient's substitute decision maker for consent to recruit/enroll patient into study	3 (6)	3 (6)
Prepare or assist in preparing study manuscripts for publication	3 (6)	8 (16)
Other: Creating study database	1 (2)	0 (0)
Other: Updating screening logs	1 (2)	0 (0)

Abbreviations: AI, artificial intelligence; CDA, confidential data agreement; CRF, case report forms; CTA, clinical trial agreement; CV, curriculum vitae; IQR, interquartile range; GCP, Good Clinical Practice; mins, minutes; MRP, most responsible physician; TCPS2, Tri-Council Policy Statement 2.

### Tasks Researchers Feel Comfortable Being Performed by AI Automation

Tasks that researchers felt comfortable allocating to AI automation are presented in Table 2. The top 5 tasks were: screening for potentially eligible patients (52%), inputting baseline and daily data into case report forms (55%), preparing internal tracking logs (51%), collecting and tracking team regulatory training documents (49%), and randomizing patients into studies (45%).

### Estimated Time to Task Completion

**Figure 3.**
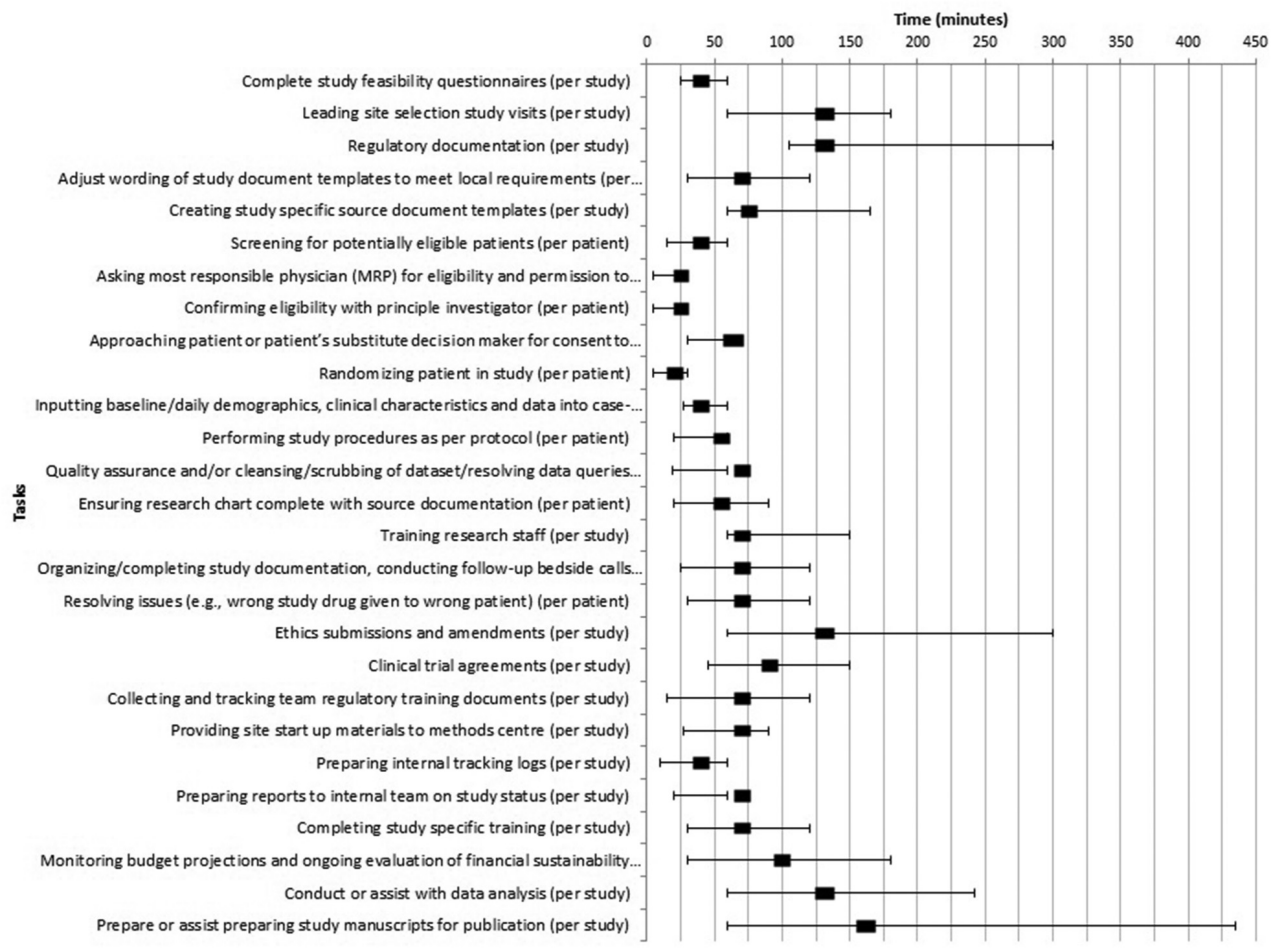
Median time (with interquartile ranges) for tasks.

Median time to task completion describe in Figure 3. Of note, tasks that could be aided by AI automation included (minutes per study patient): screening (30 min [IQR: 15-60]), randomization (10 min [IQR: 5-30]), and data collection (30 min [28-60]) which had relatively shorter estimated times to completion.

### Reallocation of Research Time With AI Automation

Respondents were asked how AI automation of some research tasks might affect their time use and research efficiency (Supplemental Figure 1). Respondents reported that they could: perform more remote data collection (64% strongly agree, 28% somewhat agree), enhance academic/community ICU recruitment (54% strongly agree, 27% somewhat agree), increase time to enroll patients (34% strongly agree, 34% somewhat agree), perform remote screening (42% strongly agree, 22% somewhat agree), and perform more remote randomization (34% strongly agree, 31% somewhat agree).

### Hospital Electronic Medical Records and Charting

Respondents were asked about charting practices and electronic medical record use in their hospitals (Supplemental Table 2).

Respondents reported that EMRs were used in their hospitals for electronic labs/investigations (65%), EMR charting (63%), electronic dictated discharge notes (53%), electronic dictated admission notes (47%), and electronic ICU flowsheets (29%). Paper charting was being used for hospital charts (53%), ICU flowsheets (47%), admission notes (41%), discharge notes (31%), paper investigations/labs (8%), and physician notes (2%).

### Open-Ended Responses

Analysis of open-ended responses provided additional context for responses to Likert scale questions (Supplemental Table 3).

Some respondents expressed comfort with AI automation: ability to perform tasks and interact with EMRs; ability to format/prepare data (with the ability for AI oversight); help with tiresome/tedious/time-consuming tasks; make processes better and more efficient; AI could perform other tasks not explicitly stated in the survey.

Some respondents expressed discomfort with aspects of AI automation: uncomfortable with certain tasks being performed by AI automation; discomfort if AI was solely performing tasks without any oversight; discomfort with tasks that require human interaction (eg, recruitment, answering patient, and family questions).

There were positive, uncertain, and negative anticipated effects of AI automation on research. Positive effects included: reduced time, increased efficiency, improved quality of work; helpful for preliminary work by AI (with appropriate oversight); reallocation of tasks to AI automation, allowing RCs/RAs to partake in tasks requiring human interaction. Uncertain themes included: uncertainty or the need for more information before AI automation implementation. Negative effects included: errors, insufficient oversight, and potential for problems; potential for inefficiencies; lack of human insight/judgment; less human interactions/involvement; research personnel job loss; safety of data security; higher costs associated with AI; and, not helpful for certain tasks.

## Discussion

In this study, Canadian ICU research personnel were asked to identify research tasks that were time-consuming, tedious, or tiresome and that might benefit from AI automation. In addition, research personnel feelings about AI automation of research tasks were assessed.

Tasks/processes that were deemed time-consuming, tedious, or tiresome and were thought to be amenable to AI automation included screening, data extraction, and randomization. Others tasks, including regulatory documentation and ethics submissions, were felt to be time-consuming or tedious/tiresome, but not amenable to AI automation.

The perceived benefits of AI automation (increased efficiency, reallocation of time for RCs to perform other tasks) must be weighed against the perceived challenges (need for AI oversight and control, loss of jobs, health regulatory legal, privacy, and proprietary implications, explanation of vision, and future of AI integration) prior to implementation in routine practice.^19,20^ There were several items (eg, data collection, screening) that some respondents felt may be possible, but may not necessarily feel comfortable with the AI performing independently. AI education for research personnel was also identified to edify on what AI capabilities and limitations are in order for AI to move forward in this research realm.

The results of this survey will be used to design a series of focus groups with AI programmers and research coordinators to help inform the design of an AI research application. This will ensure that the end-users of the AI application can provide input into the design, in order to produce the best and most user-friendly interfaces with frontline and hands-on research staff experience. The next steps would be to build a model AI program that can: (1) interface with many different types of EMRs; (2) perform tasks even after hours and on weekends; (3) increase the ability to perform research tasks remotely, thereby potentially increasing research infrastructure in community ICUs while increasing recruitment; and, (4) once established in critical care research, later expand AI automation research programs assist in other departments which perform research (eg, other medical or surgical departments, etc). We anticipate that integration of our AI application into the critical care research community will allow research endeavors to become more efficient, expand research infrastructure to current and new communities while increasing potential sample sizes and reducing time to study completion. However, it is currently unclear whether there is an effect on the overall costs of AI compared to traditional research personnel.^20^

This study has several strengths. To our knowledge, this is the first survey exploring the views of critical care healthcare researchers on AI automation. The survey was designed according to methodological recommendations,^15^ with the rigorous pilot and pretesting during the development process. Most importantly, this survey will be informative in designing the next stage of AI automation in critical care research.

The limitations of this study include the low response rate leading to possible self-selection bias, the increased number of respondents from Ontario relative to other provinces, and the overweighting of respondents from academic ICUs. Limiting to Canadian institutions was due to the perceived lack of timely research infrastructure in Canada (as shown during the Coronavirus Disease-19 pandemic), although this also limits generalizability. However, opening up the survey to other jurisdictions with similar EMR systems (eg, the United States, the United Kingdom, Australia, and New Zealand) may have further exacerbated the issue of response rate, as the denominator would be unknown in unfamiliar jurisdictions. Furthermore, many of these jurisdictions were battling outbreaks worse than Canada; hence, expecting front lines caregivers to have the bandwidth to respond to our survey may have been unrealistic. Lastly, although the use of a survey study is convenient, it may be inferior to other qualitative study designs (eg, individual interviews or focus groups) that can delve deeper into the reasons for certain responses, given the ability to ask follow-up questions.

Perhaps future work can include other jurisdictions to see what their appetite for AI automation in research will be (given the AI program's broader implication), and especially when we’re not collectively battling a pandemic). Future work could seek a few representatives from each institution to answer the survey (to maintain the diversity of responses), especially from community centers as the response rate specifically was very low. Future work could include ascertaining the acceptance of AI programs from the following groups (in addition to clinician-researchers and research personnel): public engagement, patient/family advocates, non-researcher clinicians, computer scientists (who will perform the AI programming). Furthermore, focus groups would be necessary to test and develop an eventual AI program. Future approaches could include building an AI model, running simulations and showing the data (ie, time/efficiency) and estimating costs—then obtaining some measure of acceptability, potential barriers/facilitators from respondents.

## Conclusions

In this survey study, we identified ICU research processes that were time-consuming, tedious, and/or tiresome, alongside tasks that researchers felt comfortable allocating to AI automation. Both the positive (increased efficiency and re-allocation of tasks) and negative (needing the ability for information accuracy and oversight) aspects of AI automation will need to be taken into account as AI is integrated into future research processes.

## Supplemental Material

sj-doc-1-jic-10.1177_08850666211064844 - Supplemental material for Needs Assessment Survey Identifying Research Processes Which may be Improved by Automation or Artificial Intelligence: ICU Community Modeling and Artificial Intelligence to Improve Efficiency (ICU-Comma) Click here for additional data file. Supplemental material, sj-doc-1-jic-10.1177_08850666211064844 for Needs Assessment Survey Identifying Research Processes Which may be Improved by Automation or Artificial Intelligence: ICU Community Modeling and Artificial Intelligence to Improve Efficiency (ICU-Comma) by Vincent I. Lau, Alexandra Binnie, John Basmaji, Nadia Baig, Dawn Opgenorth, Saoirse Cameron, Katie O’Hearn, Ellen McDonald, Janek Senaratne, Wendy Sligl, Danny J. Zuege, Oleksa Rewa, Sean M. Bagshaw and Jennifer Tsang in Journal of Intensive Care Medicine

sj-docx-2-jic-10.1177_08850666211064844 - Supplemental material for Needs Assessment Survey Identifying Research Processes Which may be Improved by Automation or Artificial Intelligence: ICU Community Modeling and Artificial Intelligence to Improve Efficiency (ICU-Comma) Click here for additional data file. Supplemental material, sj-docx-2-jic-10.1177_08850666211064844 for Needs Assessment Survey Identifying Research Processes Which may be Improved by Automation or Artificial Intelligence: ICU Community Modeling and Artificial Intelligence to Improve Efficiency (ICU-Comma) by Vincent I. Lau, Alexandra Binnie, John Basmaji, Nadia Baig, Dawn Opgenorth, Saoirse Cameron, Katie O’Hearn, Ellen McDonald, Janek Senaratne, Wendy Sligl, Danny J. Zuege, Oleksa Rewa, Sean M. Bagshaw and Jennifer Tsang in Journal of Intensive Care Medicine

sj-docx-3-jic-10.1177_08850666211064844 - Supplemental material for Needs Assessment Survey Identifying Research Processes Which may be Improved by Automation or Artificial Intelligence: ICU Community Modeling and Artificial Intelligence to Improve Efficiency (ICU-Comma) Click here for additional data file. Supplemental material, sj-docx-3-jic-10.1177_08850666211064844 for Needs Assessment Survey Identifying Research Processes Which may be Improved by Automation or Artificial Intelligence: ICU Community Modeling and Artificial Intelligence to Improve Efficiency (ICU-Comma) by Vincent I. Lau, Alexandra Binnie, John Basmaji, Nadia Baig, Dawn Opgenorth, Saoirse Cameron, Katie O’Hearn, Ellen McDonald, Janek Senaratne, Wendy Sligl, Danny J. Zuege, Oleksa Rewa, Sean M. Bagshaw and Jennifer Tsang in Journal of Intensive Care Medicine

sj-docx-4-jic-10.1177_08850666211064844 - Supplemental material for Needs Assessment Survey Identifying Research Processes Which may be Improved by Automation or Artificial Intelligence: ICU Community Modeling and Artificial Intelligence to Improve Efficiency (ICU-Comma) Click here for additional data file. Supplemental material, sj-docx-4-jic-10.1177_08850666211064844 for Needs Assessment Survey Identifying Research Processes Which may be Improved by Automation or Artificial Intelligence: ICU Community Modeling and Artificial Intelligence to Improve Efficiency (ICU-Comma) by Vincent I. Lau, Alexandra Binnie, John Basmaji, Nadia Baig, Dawn Opgenorth, Saoirse Cameron, Katie O’Hearn, Ellen McDonald, Janek Senaratne, Wendy Sligl, Danny J. Zuege, Oleksa Rewa, Sean M. Bagshaw and Jennifer Tsang in Journal of Intensive Care Medicine
