# Supplementary material for: Needs Assessment Survey Identifying Research Processes Which may be Improved by Automation or Artificial Intelligence: ICU Community Modeling and Artificial Intelligence to Improve Efficiency (ICU-Comma)
Source: J Intensive Care Med. 2021 Dec 13;37(10):1296–304. doi: 10.1177/08850666211064844 (PMC9468938; doi:10.1177/08850666211064844)
Supplement: sj-doc-1-jic-10.1177_08850666211064844 - Supplemental material for Needs Assessment Survey Identifying Research Processes Which may be Improved by Automation or Artificial Intelligence: ICU Community Modeling and Artificial Intelligence to Improve Efficiency (ICU-Comma) [file sj-doc-1-jic-10.1177_08850666211064844.doc]

**
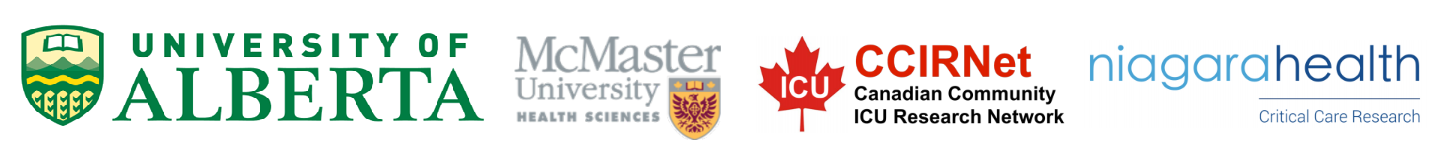
**

**Survey Questionnaire: ICU Community Modeling and Artificial Intelligence to Improve Efficiency (ICU-ComMA)**

**For Research Coordinators, Research Assistants, Research Ethics Boards Members, Health Services Officers, Clinical Information Specialist and Research Investigators**

**Needs Assessment Survey of Research Coordinator and Assistant Workflow: Optimizing Allocation of Research Coordinator Time and Identifying Inefficient Processes in Critical Care Research Which Could be Improved Through Automation or Artificial Intelligence**

**Principal Investigator**

Dr. Vincent Lau, MD, MSc, FRCPC

Assistant Professor, Clinician-Researcher

Department of Critical Care Medicine, Faculty of Medicine and Dentistry, University of Alberta

University of Alberta Hospital, Walter Mackenzie Health Sciences Centre

8440 112 Street NW, Edmonton, AB, T6G 2B7

Room C2-124 (Clinical Sciences Building)

780-407-8822 (phone)

**Co-Investigators**

Dr. Jennifer Tsang, MD, PhD, FRCPC
Associate Professor, Department of Medicine, McMaster University

Research Lead, Intensivist, Niagara Health, St. Catherine’s, ON

Dr. Alexandra Binnie, MD, DPhil, FRCPC
Intensivist, William Osler Health System, Brampton, ON

***Introduction and Purpose of Study (email invitation):***

| Subject: Needs Assessment Survey of Research Coordinator and Assistant Workflow: Optimizing Allocation of Research Coordinator Time and Identifying Inefficient Processes in Critical Care Research Which Could be Improved Through Automation or Artificial Intelligence  ICU Community Modeling and Artificial Intelligence to Improve Efficiency (ICU-ComMA)  Dear Colleague,  **Invitation to participate:** You are invited to participate in this research study about optimizing allocation of research coordinator, research assistant and investigator time during critical care research studies.  You have been invited because you are a: research coordinator, research assistants, research ethics boards members, health services officer, clinical information specialist and/or research investigators. This letter of information will explain the study and the reasons we would like your participation.  **Purpose of the study:** To perform a needs assessment survey of RCs, research assistants, research investigators and other research and information technology staff, as to what research study processes are the most time-consuming and tiresome to perform, where time allocation or inefficiencies can be improved. We will also inquire which processes or tasks would be most amenable and worthwhile investing AI automation resources into, to help improve the efficiency of a critical care research. We are also performing a needs assessment survey of research coordinators/assistants/investigators to determine which research tasks/processes could be performed by AI automation.  **Background:** In Canada, critical care research has primarily been conducted at academic tertiary care centers with access to established research infrastructure (e.g. research coordinators, assistants, etc.). However, 60-65% of healthcare in Canada is provided in community hospitals. This represents a untapped pool of ICU beds and patients which could contribute eligible patients to critical care trials that could improve efficiency with faster trial completion, and also increase critical care trial capacity in Canada. However, community ICUs often do not have established research infrastructure required to undertake critical care trials. Some of research infrastructure could be provided by artificial intelligence (AI) and automation.  There are many tasks and responsibilities that research coordinators (RC)/research assistants (RA) perform in order for patients to successfully complete a clinical trial protocol. Some tasks/processes include: (1) screen new patients against the inclusion/exclusion criteria of clinical study; (2) acquire consent from patients or their substitute decision makers to be enrolled in a clinical study; (3) assist with randomization of patients (or use of a centralized randomization database); (4) perform data extraction of vital baseline demographics and clinical characteristics before, during and after trial completion; (5) assist with quality assurance of the dataset and performing any data cleansing/scrubbing prior to locking of dataset. However, many of these daily tasks are time-consuming, potentially tedious or arduous to collect or obtain, and may not be the best utilization of a RC’s or RA’s valuable time.  **Participation:** If you wish to participate in this study, please complete the attached survey. The survey should take you approximately 10-15 minutes to complete. Once you have completed the survey, please send your responses on SurveyMonkey electronically by hitting the “submit” button. In order to minimize the risk of security breaches and to help ensure your confidentiality we recommend that you use standard safety measures such as signing out of your account, closing your browser and locking your screen or device when you are no longer using them / when you have completed the study.  Your participation is voluntary and online completion implies consent to participate.  **Benefits/Risks**: There may be some personal benefits to the study (e.g. increased efficiency) to participating in this study. Your responses to this survey are invaluable and may benefit future patients and researchers in the ICU. There are likely no potential risks to participating in this study, other than the time taken to complete this survey, with minimal risk to for your career.  **Confidentiality/Anonymity**: The information that you will share will remain strictly confidential and will be used solely for the purposes of this research. The only people who will have access to the research data are members of the research team. Your responses will be anonymous, kept confidential and only be reported in group summaries. Your answers to open-ended questions may be used verbatim in presentations and publications but neither you (nor your organization) will be identified. Individual responses will not be reported. Results will be published in pooled (aggregate) format. Anonymity is guaranteed since you are not being asked to provide your name or any personal information. You can leave the survey before submission of your answers. However once submitted, withdrawal is not possible (as data is anonymous).  **Data storage:** The final survey data will be stored by the Principle Investigator (Dr. V. Lau) on a password protected computer for 5 years. Electronic copies of the survey will be encrypted and stored on a password protected computer in the Department of Critical Care Medicine at the University of Alberta.  **Compensation:** There is no compensation for this study.  **Voluntary Participation:** You are under no obligation to participate and if you choose to participate, you may refuse to answer questions that you do not want to answer. Should you choose to withdraw midway through the electronic survey simply close the link and no responses will be included. Given the anonymous nature of the survey, once you have submitted your responses it will no longer be possible to withdraw them from the study.  **Information about Study Results**: Results of this study will be disseminated in local and national conferences. We also plan to publish in international journals as well. You may contact the principal investigator for further information or results of the study.  You may contact Dr. Vincent Lau, if you have any questions about this study. The best way to reach Dr. Lau would be through email: vince.lau@ualberta.ca.  Please keep this form for your records. Completion and submission of the survey means your consent to participate.  I appreciate your time and important contribution.  Kind regards,  Vincent Lau MD, MSc, FRCPC  The Health Research Ethics Board (HREB) at the University of Alberta in Edmonton has reviewed this study (study number: **Pro00103709)**. If you have questions about your rights as a participant, you may contact the HREB at (780) 492-2615. This office has no affiliation with the study team. All study data will be saved in SurveyMonkey in the continental USA and is subject to the US Patriot Act. All data and study documents will be stored in secured and encrypted University of Alberta cabinets and files for a minimum of 5 years following the completion of this study. SurveyMonkey questionnaire and downloaded and stored on a secure, password-protected, encrypted storage computer (on the University of Alberta server).   - Link: |
| --- |

**Questionnaire:**

1. **Please indicated your profession (choose the best answer that fits your role):**

- Clinical research coordinator
- Administrative research coordinator
- Clinical research assistant
- Research data management assistant
- Research administrative assistant
- Research ethics board members
- Research services office members
- Clinical information systems members
- Research investigator (physician)
- Patient and/or family research partner
- Other: __________________ (please specify)

1. **Please indicate your level of experience in your profession:**

- 0 – 5 years of experience
- 6 – 10 years of experience
- 11 – 15 years of experience
- 16 – 20 years of experience
- More than 20 years of experience

#

# Please indicate in which provinces you worked in the past year (select all that apply):

- Alberta (AB)
- British Columbia (BC)
- Manitoba (MB)
- New Brunswick (NB)
- Newfoundland and Labrador (NL)
- Nova Scotia (NS)
- Ontario (ON)
- Prince Edward Island (PEI)
- Quebec (QC)
- Saskatchewan (SK)
- Northwest Territories (NT)
- Nunavut (NU)
- Yukon (YT)

1. **Please indicate the size of the community in which you primarily worked in the past year:**

- Small population centres, (1,000 and 29,999 residents)
- Medium population centres, (30,000 and 199,999 residents)
- Large urban population centres (200,000 or more residents)

1. **Please indicate your primary practice setting for clinical research in the past year:**

- Teaching Hospital (e.g. confirmed teaching status by Canadian government)
- Community Hospital
- Contract research organization
- Other: __________________ (please specify)

|  | **ICU Research Processes and Tasks** |
| --- | --- |
|  |  |
|  | **As part of your research role, please indicate which tasks/processes you are involved in (check all that apply):**   - Completing study feasibility questionnaires - Leading site selection study visits (inpatient or clinic area where study will be conducted, pharmacy, labs, etc.) - Regulatory documentation (CDA, CTA, review or negotiate budget, perform or provide input in the impact analysis of the study in coordination with supporting programs (pharmacy, laboratory, etc.) - Adjust wording of study document templates (CDA, CTA, informed consents, study information letters etc.) to meet local requirements - Creating study specific source document templates - Screening for potentially eligible patients - Asking most responsible physician (MRP) for eligibility and permission to approach patients/families - Confirming eligibility with principle investigator |
|  | - Approaching patient or patient’s substitute decision maker for consent to recruit/enroll patient into study |
|  | - Randomizing patient in study |
|  | - Inputting baseline demographics, clinical characteristics and data for patient into case-report forms (CRFs) |
|  | - Inputting daily demographics, clinical characteristics and data for patient into case-report forms (CRFs) - Performing study procedures as per protocol (vital signs, drug administration, venipuncture, blood draws, etc.) - Quality assurance and/or cleansing/scrubbing of dataset/resolving data queries - Ensuring chart complete with source documentation - Training research staff - Resolving issues (e.g., wrong study drug given to wrong patient) - Ethics submissions and amendments - Clinical trial agreements - Collecting and tracking team regulatory training documents (e.g., CVs, medical license, GCP training, TCPS2 training, privacy training, etc.) - Providing site start up materials to methods centre (e.g., confirmation of training, CVS, medical license, contracts complete, delegation log, etc.) - Preparing internal tracking logs, e.g. to determine when follow-up surveys due - Preparing reports to internal team on study status - Completing study specific training (e.g., learning new data entry systems, learning each study data entry rules) - Monitoring budget projections and ongoing evaluation of financial sustainability of research projects - Conduct or assist with data analysis - Prepare or assist preparing study manuscripts for publication - Other - Not applicable |
|  |  |
|  | **On balance/average, please indicate which tasks/processes you consider time-consuming (please rate on Likert scale: 1 – not time-consuming at all, 2 – little time-consuming, 3 – somewhat time-consuming, 4 – very time-consuming, 5 – extremely time-consuming):**   - Completing study feasibility questionnaires |
|  | - Leading site selection study visits (inpatient or clinic area where study will be conducted, pharmacy, labs etc.) - Regulatory documentation (CDA, CTA, review or negotiate budget, perform or provide input in the impact analysis of the study in coordination with supporting programs (pharmacy, laboratory etc.) - Adjust wording of study document templates (CDA, CTA, informed consents, study information letters etc.) to meet local requirements - Creating study specific source document templates - Screening for potentially eligible patients - Asking most responsible physician (MRP) for eligibility and permission to approach patients/families - Confirming eligibility with principle investigator |
|  | - Approaching patient or patient’s substitute decision maker for consent to recruit/enroll patient into study |
|  | - Randomizing patient in study |
|  | - Inputting baseline demographics, clinical characteristics and data for patient into case-report forms (CRFs) Inputting daily demographics, clinical characteristics and data for patient into case-report forms (CRFs) - Performing study procedures as per protocol (vital signs, drug administration, venipuncture, blood draws, etc.) - Quality assurance and/or cleansing/scrubbing of dataset/resolving data queries - Ensuring chart complete with source documentation - Training research staff - Resolving issues (e.g., wrong study drug given to wrong patient) - Ethics submissions and amendments - Clinical trial agreements - Collecting and tracking team regulatory training documents (e.g., CVs, medical license, GCP training, TCPS2 training, privacy training, etc.) - Providing site start up materials to methods centre (e.g., confirmation of training, CVS, medical license, contracts complete, delegation log, etc.) - Preparing internal tracking logs, e.g. to determine when follow-up surveys due - Preparing reports to internal team on study status - Completing study specific training (e.g., learning new data entry systems, learning each study data entry rules) - Monitoring budget projections and ongoing evaluation of financial sustainability of research projects - Conduct or assist with data analysis - Prepare or assist preparing study manuscripts for publication - Other - Not applicable |
| 1. **On balance/average, please indicate which tasks/processes you consider tedious/tiresome (please rate on Likert scale: 1 – not tiresome at all, 2 – little tiresome, 3 – somewhat tiresome, 4 – very tiresome, 5 – extremely tiresome):**    - Completing study feasibility questionnaires | |
| - - Leading site selection study visits (inpatient or clinic area where study will be conducted, pharmacy, labs etc)   - Regulatory documentation (CDA, CTA, review or negotiate budget, perform or provide input in the impact analysis of the study in coordination with supporting programs (pharmacy, laboratory etc)   - Adjust wording of study document templates (CDA, CTA, informed consents, study information letters etc. ) to meet local requirements   - Creating study specific source document templates   - Screening for potentially eligible patients   - Asking most responsible physician (MRP) for eligibility and permission to approach patients/families   - Confirming eligibility with principle investigator | |
| - - Approaching patient or patient’s substitute decision maker for consent to recruit/enroll patient into study | |
| - - Randomizing patient in study | |
| - - Inputting baseline demographics, clinical characteristics and data for patient into case-report forms (CRFs)Inputting daily demographics, clinical characteristics and data for patient into case-report forms (CRFs)   - Performing study procedures as per protocol (vital signs, drug administration, venipuncture, blood draws, etc.)   - Quality assurance and/or cleansing/scrubbing of dataset/resolving data queries   - Ensuring chart complete with source documentation   - Training research staff   - Resolving issues (e.g., wrong study drug given to wrong patient)   - Ethics submissions and amendments   - Clinical trial agreements   - Collecting and tracking team regulatory training documents (e.g., CVs, medical license, GCP training, TCPS2 training, privacy training, etc.)   - Providing site start up materials to methods centre (e.g., confirmation of training, CVS, medical license, contracts complete, delegation log, etc.)   - Preparing internal tracking logs, e.g. to determine when follow-up surveys due   - Preparing reports to internal team on study status   - Completing study specific training (e.g., learning new data entry systems, learning each study data entry rules)   - Monitoring budget projections and ongoing evaluation of financial sustainability of research projects   - Conduct or assist with data analysis   - Prepare or assist preparing study manuscripts for publication   - Other   - Not applicable | |

| 1. **On balance/average, please indicate how much time the following tasks/processes consume, per typical patient in a typical study (in minutes):**    - Complete study feasibility questionnaires (____ minutes |
| --- |
| - - Leading site selection study visits (inpatient or clinic area where study will be conducted, pharmacy, labs etc)   - Regulatory documentation (CDA, CTA, review or negotiate budget, perform or provide input in the impact analysis of the study in coordination with supporting programs (pharmacy, laboratory etc)   - Adjust wording of study document templates (CDA, CTA, informed consents, study information letters etc. ) to meet local requirements   - Creating study specific source document templates   - Screening for potentially eligible patients   - Asking most responsible physician (MRP) for eligibility and permission to approach patients/families   - Confirming eligibility with principle investigator |
| - - Approaching patient or patient’s substitute decision maker for consent to recruit/enroll patient into study |
| - - Randomizing patient in study |
| - - Inputting baseline demographics, clinical characteristics and data for patient into case-report forms (CRFs)Inputting daily demographics, clinical characteristics and data for patient into case-report forms (CRFs)   - Performing study procedures as per protocol (vital signs, drug administration, venipuncture, blood draws, etc.)   - Quality assurance and/or cleansing/scrubbing of dataset/resolving data queries   - Ensuring chart complete with source documentation   - Training research staff   - Resolving issues (e.g., wrong study drug given to wrong patient)   - Ethics submissions and amendments   - Clinical trial agreements   - Collecting and tracking team regulatory training documents (e.g., CVs, medical license, GCP training, TCPS2 training, privacy training, etc.)   - Providing site start up materials to methods centre (e.g., confirmation of training, CVS, medical license, contracts complete, delegation log, etc.)   - Preparing internal tracking logs, e.g. to determine when follow-up surveys due   - Preparing reports to internal team on study status   - Completing study specific training (e.g., learning new data entry systems, learning each study data entry rules)   - Monitoring budget projections and ongoing evaluation of financial sustainability of research projects   - Conduct or assist with data analysis   - Prepare or assist preparing study manuscripts for publication   - Other   - Not applicable |

| 1. **As part of your research role, please indicate which tasks/processes you think could be aided/augmented by automation and/or artificial intelligence (check all that apply):**    - Complete study feasibility questionnaires |
| --- |
| - - Leading site selection study visits (inpatient or clinic area where study will be conducted, pharmacy, labs etc)   - Regulatory documentation (CDA, CTA, review or negotiate budget, perform or provide input in the impact analysis of the study in coordination with supporting programs (pharmacy, laboratory etc)   - Adjust wording of study document templates (CDA,CTA, informed consents, study information letters etc. ) to meet local requirements   - Creating study specific source document templates   - Screening for potentially eligible patients   - Asking most responsible physician (MRP) for eligibility and permission to approach patients/families   - Confirming eligibility with principle investigator |
| - - Approaching patient or patient’s substitute decision maker for consent to recruit/enroll patient into study |
| - - Randomizing patient in study |
| - - Inputting baseline demographics, clinical characteristics and data for patient into case-report forms (CRFs)Inputting daily demographics, clinical characteristics and data for patient into case-report forms (CRFs)   - Performing study procedures as per protocol (vital signs, drug administration, venipuncture, blood draws, etc.)   - Quality assurance and/or cleansing/scrubbing of dataset/resolving data queries   - Ensuring chart complete with source documentation   - Training research staff   - Resolving issues (e.g., wrong study drug given to wrong patient)   - Ethics submissions and amendments   - Clinical trial agreements   - Collecting and tracking team regulatory training documents (e.g., CVs, medical license, GCP training, TCPS2 training, privacy training, etc.)   - Providing site start up materials to methods centre (e.g., confirmation of training, CVS, medical license, contracts complete, delegation log, etc.)   - Preparing internal tracking logs, e.g. to determine when follow-up surveys due   - Preparing reports to internal team on study status   - Completing study specific training (e.g., learning new data entry systems, learning each study data entry rules)   - Monitoring budget projections and ongoing evaluation of financial sustainability of research projects   - Conduct or assist with data analysis   - Prepare or assist preparing study manuscripts for publication   - Other   - Not applicable |
| 1. **As part of your research role, what tasks/processes would you feel COMFORTABLE being aided/augmented by automation and/or artificial intelligence (check all that apply):**    - Complete study feasibility questionnaires |
| - - Leading site selection study visits (inpatient or clinic area where study will be conducted, pharmacy, labs etc)   - Regulatory documentation (CDA, CTA, review or negotiate budget, perform or provide input in the impact analysis of the study in coordination with supporting programs (pharmacy, laboratory etc)   - Adjust wording of study document templates (CDA,CTA, informed consents, study information letters etc. ) to meet local requirements   - Creating study specific source document templates   - Screening for potentially eligible patients   - Asking most responsible physician (MRP) for eligibility and permission to approach patients/families   - Confirming eligibility with principle investigator |
| - - Approaching patient or patient’s substitute decision maker for consent to recruit/enroll patient into study |
| - - Randomizing patient in study |
| - - Inputting baseline demographics, clinical characteristics and data for patient into case-report forms (CRFs)Inputting daily demographics, clinical characteristics and data for patient into case-report forms (CRFs)   - Performing study procedures as per protocol (vital signs, drug administration, venipuncture, blood draws, etc.)   - Quality assurance and/or cleansing/scrubbing of dataset/resolving data queries   - Ensuring chart complete with source documentation   - Training research staff   - Resolving issues (e.g., wrong study drug given to wrong patient)   - Ethics submissions and amendments   - Clinical trial agreements   - Collecting and tracking team regulatory training documents (e.g., CVs, medical license, GCP training, TCPS2 training, privacy training, etc.)   - Providing site start up materials to methods centre (e.g., confirmation of training, CVS, medical license, contracts complete, delegation log, etc.)   - Preparing internal tracking logs, e.g. to determine when follow-up surveys due   - Preparing reports to internal team on study status   - Completing study specific training (e.g., learning new data entry systems, learning each study data entry rules)   - Monitoring budget projections and ongoing evaluation of financial sustainability of research projects   - Conduct or assist with data analysis   - Prepare or assist preparing study manuscripts for publication   - Other   - Not applicable |

For the items above for which you indicated you would feel comfortable, please indicate why (below at end of survey):

| 1. **As part of your research role, what tasks/processes would you feel UNCOMFORTABLE being aided/augmented by automation and/or artificial intelligence (check all that apply):**    - Complete study feasibility questionnaires |
| --- |
| - - Leading site selection study visits (inpatient or clinic area where study will be conducted, pharmacy, labs etc)   - Regulatory documentation (CDA, CTA, review or negotiate budget, perform or provide input in the impact analysis of the study in coordination with supporting programs (pharmacy, laboratory etc)   - Adjust wording of study document templates (CDA,CTA, informed consents, study information letters etc. ) to meet local requirements   - Creating study specific source document templates   - Screening for potentially eligible patients   - Asking most responsible physician (MRP) for eligibility and permission to approach patients/families   - Confirming eligibility with principle investigator |
| - - Approaching patient or patient’s substitute decision maker for consent to recruit/enroll patient into study |
| - - Randomizing patient in study |
| - - Inputting baseline demographics, clinical characteristics and data for patient into case-report forms (CRFs)Inputting daily demographics, clinical characteristics and data for patient into case-report forms (CRFs)   - Performing study procedures as per protocol (vital signs, drug administration, venipuncture, blood draws, etc.)   - Quality assurance and/or cleansing/scrubbing of dataset/resolving data queries   - Ensuring chart complete with source documentation   - Training research staff   - Resolving issues (e.g., wrong study drug given to wrong patient)   - Ethics submissions and amendments   - Clinical trial agreements   - Collecting and tracking team regulatory training documents (e.g., CVs, medical license, GCP training, TCPS2 training, privacy training, etc.)   - Providing site start up materials to methods centre (e.g., confirmation of training, CVS, medical license, contracts complete, delegation log, etc.)   - Preparing internal tracking logs, e.g. to determine when follow-up surveys due   - Preparing reports to internal team on study status   - Completing study specific training (e.g., learning new data entry systems, learning each study data entry rules)   - Monitoring budget projections and ongoing evaluation of financial sustainability of research projects   - Conduct or assist with data analysis   - Prepare or assist preparing study manuscripts for publication   - Other   - Not applicable |

For the items above for which you indicated you would feel uncomfortable, please indicate why (below at end of survey):

|  |
| --- |
|  |
|  |
|  |
|  |

| 1. **Please indicate the rating which indicates your feelings best for the following questions (please rate on Likert scale: 1 – strongly disagree, 2 – somewhat disagree, 3 – neutral, 4 – somewhat agree, 5 – strongly agree, 6 - not applicable, 7 – prefer not answering, 8 – unsure):**    1. **Overall, do you feel automation or artificial intelligence would better allocate your time in research, perhaps freeing up your time to do consent or recruitment tasks/processes?**    2. **Overall, if automation or artificial intelligence could assist you in your job, do you feel you could recruit more patients per day?**    3. **Overall, if automation or artificial intelligence could assist you in your job, do you feel you could perform more remote screening, randomization and data collection?**    4. **Overall, if automation or artificial intelligence could assist you in your job, do you feel recruitment in academic/community ICUs could be enhanced?**    5. **As a research ethics board member, do you feel automation or artificial intelligence would be feasible in performing research tasks?**    6. **As a research ethics board member, do you feel automation or artificial intelligence can be used ethically to perform research tasks?**    7. **As a patient or family partner in research, do you feel automation or artificial intelligence would be feasible in performing research tasks?**    8. **As a patient or family partner in research, do you feel automation or artificial intelligence can be used ethically to perform research tasks?** |
| --- |
|  |
| 1. **Currently, what are the information technology and charting practices at your site? (select all that apply)** |
| - - Electronic charting (on electronic medical record)   - Paper hospital charts |
| - - Electronic dictated admission notes (on electronic medical record)   - Paper admission notes   - Electronic dictated discharge notes (on electronic medical record)   - Paper discharge notes   - Electronic labs/investigations (on electronic medical record)   - Paper labs/investigations   - Electronic ICU flowsheet (on electronic medical record) |
| - - Paper ICU flowsheet |
| - - Not applicable   - Prefer not answering   - Unsure   **What positive benefits do you anticipate from using automation/artificial intelligence (AI) to help with research coordination?**  **What negative effects do you anticipate from using AI to help with research coordination?** |

**Any other feedback on this topic? (free-text)**

**We would like to thank you very much for your participation and your opinions.**
