# Supplementary material for: Needs Assessment Survey Identifying Research Processes Which may be Improved by Automation or Artificial Intelligence: ICU Community Modeling and Artificial Intelligence to Improve Efficiency (ICU-Comma)
Source: J Intensive Care Med. 2021 Dec 13;37(10):1296–304. doi: 10.1177/08850666211064844 (PMC9468938; doi:10.1177/08850666211064844)
Supplement: sj-docx-2-jic-10.1177_08850666211064844 - Supplemental material for Needs Assessment Survey Identifying Research Processes Which may be Improved by Automation or Artificial Intelligence: ICU Community Modeling and Artificial Intelligence to Improve Efficiency (ICU-Comma) [file sj-docx-2-jic-10.1177_08850666211064844.docx]

Supplemental Table 1. Tasks/processes involved in researcher role (n = 49)

| Task | Responses (%) |
| --- | --- |
| Ethics submissions and amendments | 41 (84) |
| Resolving issues (e.g., wrong study drug given to wrong patient) | 36 (73) |
| Adjust wording of study document templates (CDA, CTA, informed consents, study information letters etc.) to meet local requirements | 35 (71) |
| Creating study specific source document templates | 35 (71) |
| Screening for potentially eligible patients | 35 (71) |
| Training research staff | 35 (71) |
| Prepare or assist preparing study manuscripts for publication | 35 (71) |
| Regulatory documentation (CDA, CTA, review or negotiate budget, perform or provide input in the impact analysis of the study in coordination with supporting programs (pharmacy, laboratory, etc.) | 34 (70) |
| Quality assurance and/or cleansing/scrubbing of dataset/resolving data queries | 33 (67) |
| Preparing internal tracking logs, e.g. to determine when follow-up surveys due | 33 (67) |
| Completing study specific training (e.g., learning new data entry systems, learning each study data entry rules) | 33 (67) |
| Approaching patient or patient’s substitute decision maker for consent to recruit/enroll patient into study | 32 (65) |
| Ensuring research chart complete with source documentation | 32 (65) |
| Preparing reports to internal team on study status | 32 (65) |
| Inputting baseline demographics, clinical characteristics and data for patient into case-report forms (CRFs) | 31 (63) |
| Organizing and completing study documentation for enrollment, randomization, and consent procedures to your list and informing/teaching nurse staff of study procedures and tests being done and conducting follow-up bedside calls to ensure study procedures are being followed | 31 (63) |
| Monitoring budget projections and ongoing evaluation of financial sustainability of research projects | 31 (63) |
| Asking most responsible physician (MRP) for eligibility and permission to approach patients/families | 30 (61) |
| Inputting daily demographics, clinical characteristics and data for patient into case-report forms (CRFs) | 30 (61) |
| Collecting and tracking team regulatory training documents (e.g., CVs, medical license, GCP training, TCPS2 training, privacy training, etc.) | 30 (61) |
| Confirming eligibility with principal investigator | 30 (61) |
| Conduct or assist with data analysis | 29 (59) |
| Randomizing patient in study | 28 (57) |
| Providing site start up materials to methods centre (e.g., confirmation of training, CVS, medical license, contracts complete, delegation log, etc.) | 27 (55) |
| Completing study feasibility questionnaires | 25 (51) |
| Clinical trial agreements | 25 (51) |
| Leading site selection study visits (inpatient or clinic area where study will be conducted, pharmacy, labs, etc.) | 23 (47) |
| Performing study procedures as per protocol (vital signs, drug administration, venipuncture, blood draws, etc.) | 21 (43) |
| Other: Grant applications | 2 (4) |
| Other: Creating study databases | 1 (2) |
| Other: Hiring new staff | 1 (2) |
| Other: Organizing research meetings | 1 (2) |
| Other: Creating and editing research-related education modules for frontline staff | 1 (2) |
| Other: Study oversight (regulatory approvals, organization, implementation, closure) | 1 (2) |
| Other: Study reports (for funding agencies) | 1 (2) |
| Not applicable | 1 (2) |

CDA = confidential data agreement, CRF = case report forms, CTA = clinical trial agreement, CV = curriculum vitae, IQR = interquartile range, GCP = Good Clinical Practice, mins = minutes, MRP = most responsible physician, TCPS2 = Tri-Council Policy Statement 2
