# Supplementary material for: Needs Assessment Survey Identifying Research Processes Which may be Improved by Automation or Artificial Intelligence: ICU Community Modeling and Artificial Intelligence to Improve Efficiency (ICU-Comma)
Source: J Intensive Care Med. 2021 Dec 13;37(10):1296–304. doi: 10.1177/08850666211064844 (PMC9468938; doi:10.1177/08850666211064844)
Supplement: sj-docx-3-jic-10.1177_08850666211064844 - Supplemental material for Needs Assessment Survey Identifying Research Processes Which may be Improved by Automation or Artificial Intelligence: ICU Community Modeling and Artificial Intelligence to Improve Efficiency (ICU-Comma) [file sj-docx-3-jic-10.1177_08850666211064844.docx]

Supplemental Table 2. Information technology and charting practices (n = 49)

| Task | Positive response (%) |
| --- | --- |
| Electronic labs/investigations (on electronic medical record) | 32 (65) |
| Electronic charting (on electronic medical record) | 31 (63) |
| Paper hospital charts | 26 (53) |
| Electronic dictated discharge notes (on electronic medical record) | 26 (53) |
| Electronic dictated admission notes (on electronic medical record) | 23 (47) |
| Paper ICU flowsheet | 23 (47) |
| Paper admission notes | 20 (41) |
| Paper discharge notes | 15 (31) |
| Electronic ICU flowsheet (on electronic medical record) | 14 (29) |
| Unsure | 5 (10) |
| Paper labs/investigations | 4 (8) |
| Paper physician notes | 1 (2) |
| Prefer not answering | 1 (2) |

ICU = intensive care unit
