# Supplementary material for: Needs Assessment Survey Identifying Research Processes Which may be Improved by Automation or Artificial Intelligence: ICU Community Modeling and Artificial Intelligence to Improve Efficiency (ICU-Comma)
Source: J Intensive Care Med. 2021 Dec 13;37(10):1296–304. doi: 10.1177/08850666211064844 (PMC9468938; doi:10.1177/08850666211064844)
Supplement: sj-docx-4-jic-10.1177_08850666211064844 - Supplemental material for Needs Assessment Survey Identifying Research Processes Which may be Improved by Automation or Artificial Intelligence: ICU Community Modeling and Artificial Intelligence to Improve Efficiency (ICU-Comma) [file sj-docx-4-jic-10.1177_08850666211064844.docx]

**Supplemental Table 3.** Thematic content analysis of open-ended responses

| Themes | | | Theme Illustrative Quotes |
| --- | --- | --- | --- |
| Researchers comfort with tasks performed by automation/artificial intelligence (AI) | | | |
| Comfortable | **Administration** | *“Many of the items could be made more efficient through AI systems linked to electronic medical records that can identify patients, pull baseline data, ask most responsible physician for agreement, ensure all data collected, do data analysis.”*  *“Algorithms can be generated to complete data collection”*  *“Direct information extraction - no need for application of clinical knowledge-skills* | |
|  | **Multi-tasking** | *“Some of these tasks could be formatted or prepared automatically with final review by research team.”*  *“There's the ability to cross check and use what the AI creates”*  *“I think that the AI would be able to provide a basis upon which I could simply review and confirm to be accurate thus saving a great deal of time.”*  *“There are many features of the processes that could be standardized. All would need to be reviewed for accuracy/legitimacy.”*  *“Specifically the internal tracking logs, this is already automatically done for some studies but it would be nice if there was a one system that could do this for all studies.”* | |
|  | **Efficiency** | *“The procedure is repetitive and therefore could be done efficiently through automation.”*  *“Straight-forward, repetitive, low risk of errors, reduce bias”* | |
| Uncomfortable & perceived challenges | **Lack of clinical and human judgment** | *“I feel uncomfortable with AI automation performing the following tasks: …site selection, manuscript preparation…anything regarding patient safety or oversight…in-person study starts training at new centres…approaching families [or] confirmation of eligibility…accounting [or] resolving problems/issues…submission of regulatory documents to research ethics boards for my trial…consent, randomization, data cleaning/analysis…baseline data/vitals…quality assurance, ethics…clinical trial applications, research ethics boards applications, preparing source documents…training…”* | |
|  | **Lack of oversight** | *“Generally, I would feel uncomfortable with allowing a completely automated or AI process for any task that relied solely on automation and/or AI, without a process for me to check the final result. So, for example, I would be comfortable for an automated process for randomization and screening; but I would likely still want some sort of process where I would review to make sure that everything is captured”*  *“Comfortable having assistance with screening, data entry, etc. but would have to confirm it myself.”*  *“I would like to be able to double-check aspects of the work that is being aided by automation (e.g., I would review any internal reports generated by AI to ensure accuracy; I would confirm eligibility myself if a potential patient was flagged as being potentially eligible by AI).  “* | |
|  | **Lack of human interaction** | *“Consent discussions are very personal and an opportunity to support patients/families and build rapport. Would not feel comfortable delegating this task to AI. Confirmation of study eligibility requires clinical knowledge of contraindications, appropriateness, and advanced judgment based on experience. Would be comfortable being aided by AI but not the final confirmation.”*  *“Approaching patients; making person to person contact as this is something that's unique in the sense that families are going through something emotional and having the ability to adapt approach”*  *“Tasks that typically involve human interaction and rapport building should not be done by AI as it's important to build a relationship with participants and clinical staff. Also, tasks that involve fine-tuned attention to detail such as in data or in editing/wording are not as ideal for automation/AI.”*  *“Discussion with most responsible physician/nurse, confirming eligibility with principal investigator…consenting…any patient/family interactions.”*  *“Anything that has to do with meeting/interacting with potential patients/families…’big picture’ tasks - such as site visits”*  *“I find that as a study is activated on site and we start enrolling patients into the study, there are often nuanced situations that arise that require attention from someone well versed in research/clinical setting.”* | |
| Effects of automation/AI | | | |
| Positive themes | **Efficiency** | *“Reduce the time spent on tedious tasks to allow more time spent to other priorities. This would enable projects to progress faster.”*  *“Simplify task and help reach clinical team or keep in touch.”*  *“I think it could make certain screening, recruiting and data collection processes much more efficient and reduce the burden on coordinators, hospital site leads, and patients/their families by decreasing unnecessary interactions and workload.”*  *“Saving time and cost - improve efficiency.”*  *“Automation of electronic based tasks would save time of tasks that require tedious editing.”*  *“Increased identification of patients, decreased time spent entering data.”*  *“Improved efficiency. Fewer patients missed. Less time-consuming, inefficient labour. “*  *“Reduced bias may save time with repetitive/tedious tasks.”*  *“Efficiency, speed, accuracy, objectivity, impartiality (which can also be a disadvantage in some cases).”*  *“Increase data quality for points that have direct extraction.”*  *“I think AI could benefit us most in the following 3 areas: 1) screening for potentially eligible patients, 2) data collection, and 3) organization of study documents. Automated data collection may reduce human error in data collection if programmed correctly and audited periodically. AI would also help free up some of my time and allow for richer conversations with patients/families during consent/follow-up and more time to develop training/information documents for patients/families/ICU colleagues and more time to foster a research culture in the ICU.”* | |
|  | **AI oversight** | *“I think AI would be very helpful in doing preliminary screening (perhaps based on some quick eligibility flags), abstracting data (especially straight forward data; e.g., blood work, culture results, etc.; may even reduce human error), entering data into paper/electronic case-report forms, and keeping tabs on regulatory documents / letting the team know when certifications need to be updated, etc. A double-check/oversight feature should still be included but this would help free up time for other more complex research tasks.”* | |
|  | **Resource allocation and human interaction** | *“Better quality of data collection and time spent on tasks where a human being is absolutely necessary (consent, etc.)”*  *“Being able to assist with decision making/organization that cannot be done simultaneously when working on multiple projects.”*  *“I think it will make recruitment and data entry much easier. When I come in at work, I spend a lot of my time recruiting patients so if that process can be automated, that would give me more time to work on my other tasks.”*  *“For our specific hospital, I think AI could help with some of the more tedious tasks and free up more time for manuscript writing/grant applications/scholarly output. In a pediatric ICU with only 10 beds, lack of time is not really an issue that limits recruitment (maybe on weekends for the studies that we do not have someone on call). So the benefit for us for AI would not be recruitment but I think it could help our group to be more productive overall but allowing us to focus our attention on other tasks. For community hospitals, or even academic hospitals that do not have a full time RA/RC for given study, I think that AI could help with recruitment.”*  *“The opportunity to use AI to assist with some of the more time consuming, tedious tasks which would free up time for recruitment/consent discussions and follow-up visits with patients/families.”*  *“Freeing up time to do the more hands on research required - interact with families, teaching.”*  *“Increased efficiency to be able to more easily identify eligible patients for our studies, in particular, with limited human resources.”*  *“It could save time for research coordinator and staff to do other tasks.”* | |
| Unclear or uncertain themes | **Education and uncertainty** | *“I would need to know what the vision of AI was to be able to say whether it would work or not. Possibly help with screening but I am not sure beyond that.”*  *“I don't know enough about it to comment.”*  *“Remains to be proven if it enhances efficiency and reduces duplication.”*  *“No concerns - need more information about how it would work to full understand risks”* | |
| Negative themes | **Data quality** | *“Programming errors, insufficient oversight”*  *“The possibility of errors due to automation. Less chance to identify irregularities which could arise. For example if patient eligibility was automated, you may not know to program all unique situations which could affect eligibility.”*  *“There are often exceptions to the rules in research and recruitment, which may require a human to make a decision that AI couldn't.”*  *“Systemic errors due to poor coding.”*  *“Complexity of systems means that they will require a lot of support in the event of problems.”*  *“Still requires human oversight, time needed in troubleshooting, figuring out what went wrong early.”*  *“May be difficult to trace glitches if adjustments needed after start of production.”*  *“We still will have to review some AI completed tasks to ensure they are accurate.”*  *“If there are issues with data outputs and it went undetected.”*  *“Nuanced items could get missed by automation.”*  *“Unsure about attention to detail or overseeing work from AI.”* | |
|  | **Inefficiency** | *“In the beginning, there would be a learning/confirmation period to ensure that the AI is reliable in the tasks that it is doing; which would be more time consuming for research staff.”*  *“Remains to be proven if it enhances efficiency and reduces duplication.”*  *“I also think the learning curve might be steep, especially if the research team and the unit do not fully support the system.”*  *“The time it would take to set up the system.”* | |
|  | **Lack of clinical and human judgment** | *“The algorithms may not be flexible and there is no clinical judgment.”*  *“This depends on what aspects of research AI would take over. For example, if AI took over screening, I imagine I would know less about the patients myself, which would make conversations with MRP more difficult. I would not be 100% convinced that I am ensuring patient safety when enrolling patients into a study, if I have not looked through their notes and chart myself.”*  *“I think AI could assist but not replace clinical/research expertise and judgment. Without oversight, there is the potential for errors to accumulate.”*  *“Potential loss of control of processes that require complicated decision-making.”* | |
|  | **Lack of human interactions** | *“Maybe inability to get feel from direct bedside staff as to their level of comfort with possible enrolments or continuation of trial interventions.”*  *“Worry that it is impersonal and perhaps inaccurate under certain conditions.”*  *“Decreased principal investigator engagement in study details.”*  *“Potential for dehumanization of processes.”* | |
|  | **Automation driven job loss** | *“In some cases, it could lead to job loss (or not renewing contracts). For example, in our centre as the research coordinator, I do more big picture tasks (clinical trial applications, research ethics boards manuscripts, etc.) and our research assistant mostly does the tasks I flagged as "tedious" or "could be done by AI". If AI was able to take over a lot of these tasks, there might not be enough work for her to renew the contract. Other centres might have similar situations.”* | |
|  | **Confidentiality** | *“There is always the possibility of error and with any type of data sharing/mining, there is always a threat to data security.”*  *“Issues pertaining to breeches in patient confidentiality.”* | |
|  | **Cost-effective** | *“Costs of operation”* | |
|  | **Not helpful for certain tasks** | *“Not useful for many of the day to day tasks.”* | |
| Feelings/opinions about automation/AI | | | |
| Positive themes | **Advancement of research** | *“We started process of using electronic live charts for study recruiting, follow-up tracking, online database population, tracking of study - and other relevant documents, separate data analysis and preparation for publication in DETECT study in multi-site trial in 2016 with great success. Only one site investigator and one study coordinator per site was needed to conduct the entire study. We had full access to an electronic patient chart and ORACLE database with web interface, allowing data entry, reporting, collection of documents, follow-up tracking in live reporting. It was one of the most effective trials I was ever involved in.”*  *“AI in clinical research is worth exploring.”*  *“I strongly support better automation and educating people on how to implement automation into their research practices as I often feel that researchers and institutions tend to get stuck in their ways of doing things and it's a lot of time lost.”*  *“This is really a good idea! Since AI will be the leading industry in the upcoming years.”*  *“If AI could automatically pull from electronic charts and populate CRFs, this would be hugely helpful. Flexibility in adjusting settings for AI data collection for different studies would be needed.”* | |
| Balanced, neutral or uncertain themes | **Reservation or uncertainty** | *“Overall, AI would be very helpful but would need to have some oversight and designed/programmed well in consultation with clinical staff to better understand the parameters of the tasks and allow for appropriate flagging of things that may need to be double-checked/verified by a human.”*  *“I think AI can be very powerful and can definitely help me do my job more efficiently but there is also a place for human decision-making and judgment which cannot be replaced by AI, so it is important to carefully consider which aspects of the job we want/and would feel comfortable to automate.”*  *“It is difficult to anticipate the benefit of AI as I do not see how it would be functional at our site. For sites where everything is electronic, I feel it would be more valuable. At best, AI could flag potential patients, but they would not be fully able to screen a patient based on paper charting practices.”* | |
| Negative themes | **Archaic practices** | *“Definitely need an up to date electronic charting system to go AI.”* | |
